# Functionalized core-shell hydrogel microsprings by anisotropic gelation with bevel-tip capillary

**DOI:** 10.1038/srep45987

**Published:** 2017-04-05

**Authors:** Koki Yoshida, Hiroaki Onoe

**Affiliations:** 1 Center for Multidisciplinary and Design Science, Graduate School of Integrated Design Engineering, Keio University, 3-14-1 Hiyoshi, Kohoku-ku, Yokohama 223-8522, Japan

## Abstract

This study describes a novel microfluidic-based method for the synthesis of hydrogel microsprings that are capable of encapsulating various functional materials. A continuous flow of alginate pre-gel solution can spontaneously form a hydrogel microspring by anisotropic gelation around the bevel-tip of the capillary. This technique allows fabrication of hydrogel microsprings using only simple capillaries and syringe pumps, while their complex compartmentalization characterized by a laminar flow inside the capillary can contribute to the optimization of the microspring internal structure and functionality. Encapsulation of several functional materials including magnetic-responsive nanoparticles or cell dispersed collagen for tissue scaffold was demonstrated to functionalize the microsprings. Our core-shell hydrogel microsprings have immense potential for application in a number of fields, including biological/chemical microsensors, biocompatible soft robots/microactuators, drug release, self-assembly of 3D structures and tissue engineering.

Spring structures are ubiquitous in living organisms because of their unique properties (especially at the microscale). For example, spring-shaped bodies of *Flagella* and *Spirochetes*^1^,^2^ allow them to efficiently move in liquids in accordance with Stokes’ law in viscous fluids. Vorticella^3^,^4^ can rapidly travel large distances by using their stalks as soft and flexible spring-shaped living actuators, while various receptors, such as Meissner’s corpuscles^5^, play roles of soft tactile sensors. Hence, the described organs realize their primary functions because of the presence of microspring-shaped internal actuating/sensing components.

From an engineering point of view, these microsprings and their unique properties can be possibly used in microscale mechanical systems. For mimicking such spring structures functional polymers can serve as effective materials. In particular, hydrogels possess high mechanical flexibility and capability for encapsulating functional materials, and thus can exhibit stimulus responsiveness^6^,^7^ and programmability^8^,^9^ after proper functionalization. As a result, they can be utilized in biocomputing^10^, scaffolds for cell culture^11^,^12^, chemical reaction-based actuators^13^,^14^, chemical/biological sensors^15^,^16^, and drug delivery systems^17^. Several methods for producing microsprings from hydrogels have been reported, including two-photon stereo-lithography^18^, shrinkage volume difference^19^, liquid rope coiling^20^ and anisotropic swelling^21^ which resulted in the formation of a microspring array, double-layered microspring, core-shell microspring and thermally-responsive ring, respectively. However, these hydrogel-based microsprings were composed of single hydrogel materials. Furthermore the formation of heterogeneous hydrogel microsprings possessing the ability to encapsulate various functional materials has not yet been achieved.

In this work, we describe a simple and versatile microfluidic-based method for fabricating hydrogel microsprings that are capable of encapsulating functional materials. We use anisotropic gelation at a bevel-tip capillary for hydrogel microspring formation, whose mechanism is different from those described in previous studies^18^,^19^,^20^. After extrusion of a sodium alginate pre-gel solution into a calcium chloride solution via a bevel-tip capillary, the alginate pre-gel solution in vicinity of the tip anisotropically gelates, thus a calcium alginate hydrogel microspring structure is continuously formed (Fig. 1a, Movie 1, detailed spring constant is in Supplementary Information S3). This method allows the fabrication of hydrogel microsprings using only simple capillaries and syringe pumps; however, it also aids in encapsulating various functional materials by creating a coaxial laminar flow (Fig. 1b). In this study, we report the optimal spring fabrication conditions and elucidate a possible formation mechanism. Then, we demonstrate the formation of heterogeneous core-shell hydrogel microsprings that are capable of encapsulating functional materials with various properties.

## Formation of hydrogel microsprings

**Figure 1:**
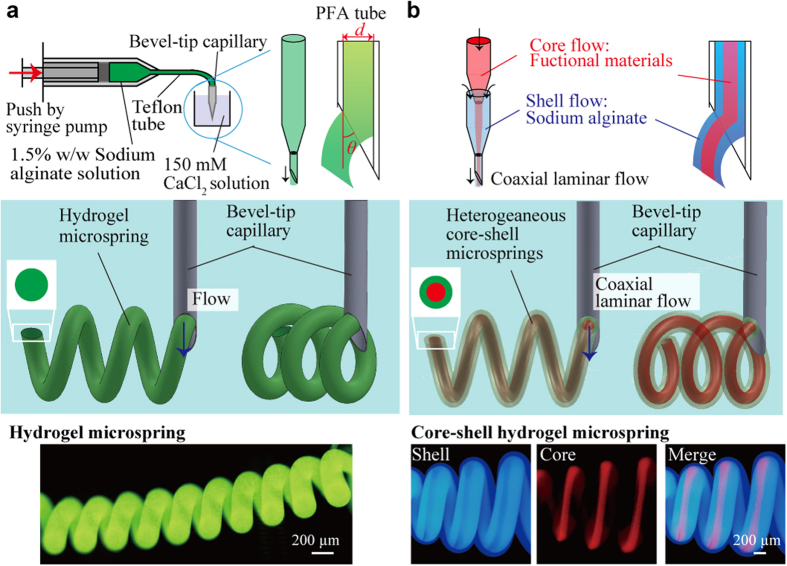
Formation of a hydrogel microspring with a bevel-tip capillary. (**a**) Schematics showing the fabrication setup and images of the produced hydrogel microsprings. A calcium alginate hydrogel microspring is continuously formed by extruding the sodium alginate pre-gel solution into the calcium chloride solution using a bevel-tip capillary. The obtained fluorescent image confirmed that a stable hydrogel microspring with more than 10 turns, outer diameter of 500 μm, and length of 3 mm was spontaneously fabricated. (**b**) A heterogeneous core-shell microspring fabricated by creating a coaxial laminar flow in the bevel-tip capillary. The corresponding fluorescent images contain the distinct core (red) and shell (blue) parts.

In order to produce hydrogel-based microsprings (Fig. 1a, top), a bevel-tip capillary was prepared by cutting a perfluoroalkoxy (PFA) microtube with the inner diameter *d* = 100–300 μm into smaller pieces (the magnitude of the tip angle *θ* was adjusted to 20–90°). The obtained capillary was connected to a syringe filled with a 1.5% w/w sodium alginate solution. Using a syringe pump, the sodium alginate solution was then extruded at a constant flow rate into a 150 mM CaCl_2_ solution. Since the flow of the sodium alginate solution was characterized by a low Reynolds number (Re < 0.5), a laminar flow was formed inside the capillary (Fig. 1a, middle), and the sodium alginate solution instantly gelated by Ca^2+^ ions near the beveled tip. The fluorescent image (Fig. 1a, bottom) indicates the spontaneous fabrication of a stable hydrogel microspring with more than 10 turns, outer diameter of 500 μm, and length of 3 mm. Using the described method, hydrogel microsprings with outer diameters varying from 188 μm to 2790 μm were successfully produced (Figure S3). Maximum stable length of a hydrogel microspring was approximately 20 turns because the hydrogel microspring was bent by gravity when the length of the hydrogel microspring became longer. In addition, the cross-sectional pattern of the laminar flow can be easily adjusted by modifying the design of the microfluidic channel. Using the device described in Figure S1 and Table S1, a coaxial laminar flow can be created in the capillary (Fig. 1b) and then extruded into the CaCl_2_ solution to produce a core-shell hydrogel microspring (Fig. 1b, bottom). The corresponding fluorescence microscopy image revealed that the fabricated microspring contained distinct inner and outer parts.

**Figure 2:**
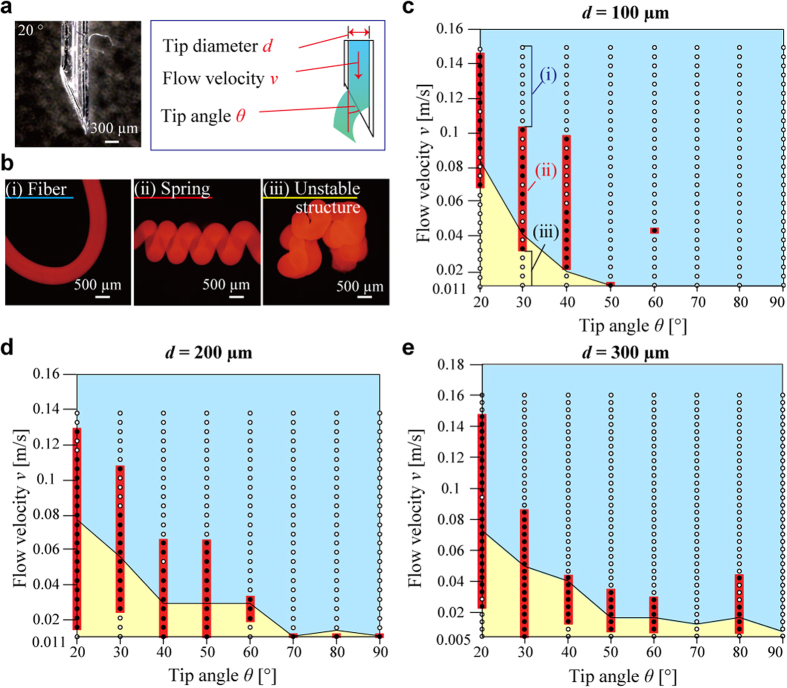
Conditions utilized during manufacturing of hydrogel microsprings. (**a**) Parameters of the hydrogel microspring formation. (**b**) Three types of the fabricated hydrogel structures: (i) a fiber, (ii) a spring, and (iii) an unstable structure. (**c**–**e**) Success/failure diagrams of the hydrogel microspring formation obtained by varying the tip angle *θ* and the flow velocity *v* for each tip diameter *d*. The solid circles (highlighted by the red lines) denote the successful formation of springs, and the open circles indicate that fibers or unstable structures were produced instead. The blue and yellow regions correspond to the formation of fibers and unstable structures, respectively.

Successful formation of hydrogel microsprings depends on various parameters, including capillary (tip diameter, tip angle, and surface wettability), fluidic (flow velocity, viscosity, and density), and reaction (temperature and solution concentration) ones. To obtain the optimal conditions for the hydrogel microspring fabrication, the following three parameters, which did not affect the course of the chemical reaction, were varied: the tip angle *θ*, tip diameter *d*, and flow velocity *v* (Fig. 2a). To evaluate the success or failure of the spring formation, the hydrogel microstructures produced using the bevel-tip capillary were divided into the three main types: fibers (Fig. 2b(i), Movie 2), springs (Fig. 2b(ii), Movie 3), and unstable randomly bent or bulk structures (Fig. 2b(iii), Movie 4). A relationship between the flow velocity *v* (0.011–0.18 m/s) and the tip angle *θ* (20–90°) was plotted for each tip diameter *d* (100–300 μm; see Fig. 2c–e). In particular, the described fabrication procedure was performed five times at each condition (its detailed description is provided in the Supporting Information section S4, Figure S3), and the depicted solid circles (highlighted with the red lines) corresponded to the successful formation of a spring (>20% success ratio). The described stochastic method for estimating the success ratio of the spring formation was utilized because the spring formation process was affected by various instability factors of the experimental system, such as the timing of injection the bevel-tip capillary into calcium chloride solution. In addition, when a spring was not formed, either a fiber or an unstable structure was produced (Information section S6, Figure S5); the corresponding conditions are denoted as the “fiber region” and the “unstable structure region” in blue and yellow in the obtained plot, respectively.

All the plotted graphs (Fig. 2c–e; the detailed data are presented in the Supporting Information section S7, Figure S6) indicated the fiber formation at high flow velocities (Fig. 2c–e, blue areas), while unstable structures were formed at low flow velocities (Fig. 2c–e, yellow areas). It was also found that the successful spring formation was observed in the areas spanning between the fiber and the unstable structure regions (Fig. 2c–e, red bars) at all tip diameters (*d* = 100–300 μm). The increase in the tip angle *θ* from 20° (steep tip) to 90° (flat tip) narrowed the flow velocity range corresponding to the successful spring formation. The boundary of the flow velocity shifted to lower numbers, regardless of the tip diameter. Spring formation rarely occurred at *θ* > 40°, indicating that the sharp bevel tip (corresponding to *θ* < 30°) was important for stable spring fabrication.

In the next step, the scale effect of the utilized capillaries was investigated at all tip diameters (*d* = 100–300 μm; see Fig. 2c–e). The maximum flow velocity of the produced springs decreased with an increase in the tip angle *θ* from 20° (steep tip) to 90° (flat tip). At the tip angles *θ* = 20° and 30°, the maximum spring flow velocity (Fig. 2c–e, red bars) was approximately the same, regardless of the tip diameter (*v* = 0.127–0.146 m/s at *θ* = 20° and 0.085–0.106 m/s at *θ* = 30°). In addition, the boundary flow velocity between the fiber regions (Fig. 2c–e, blue areas) and the unstable structure regions (Fig. 2c–e, yellow areas) also decreased with an increase in the tip angle *θ* from 20° to 90°. At the tip angles *θ* = 20° and 30°, the values of the boundary flow velocity were also approximately the same (*v* = 0.075–0.080 m/s at *θ* = 20° and 0.037–0.058 m/s at *θ* = 30°). Hence, the magnitudes of the maximum and boundary flow velocities remained constant at different tip diameters within the range of *d* = 100–300 μm.

### Size variety of hydrogel microsprings

**Figure 3:**
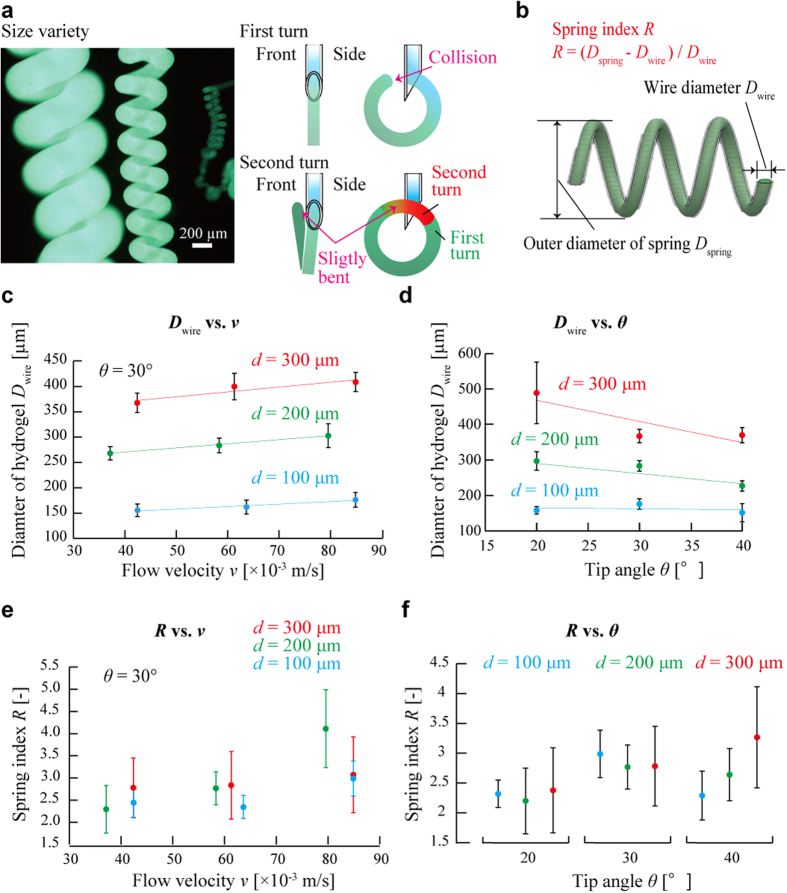
Size variety of hydrogel microsprings. (**a**) Microsprings of various sizes fabricated using capillaries with different tip diameters and an image of forming densely packed hydrogel microsprings. (**b**) Microspring shape parameters. (**c**,**d**) Shape analysis of the fabricated hydrogel microsprings performed by varying the flow velocity, tip angle, and tip diameter.

The shapes of the hydrogel microsprings produced under different conditions were examined as well (Fig. 3a left). In particular, the effects of the tip diameter *d*, tip angle *θ*, and flow velocity *v* on the resulting microspring shape have been investigated. The pitches of the hydrogel microsprings were mostly densely packed because the spring was slightly bent by collision to the capillary at the first turn (Fig. 3a right). The wire diameter *D*_wire_, outer diameter of spring *D*_spring_, and spring index *R* (which was used as an indicator of the spring strength^22^ and was defined by the expression *R* = (*D*_spring_ − *D*_wire_)/*D*_wire_ (Fig. 3b)) were estimated first in the tip angle range *θ* = 20–40° at a specified value of the flow velocity *v* (see Table S4) and then in the flow velocity range *v* = 0.030–0.090 m/s at a constant tip angle *θ* of 30°. The wire diameter *D*_wire_ was mainly dependent on the tip diameter *d*, proportional to the flow velocity *v*, and inversely proportional to the tip angle *θ* (see Fig. 3c,d; the estimated contributions of *d (D*_wire_ vs. *v*), *d (D*_wire_ vs. *θ*), *v*, and *θ* were 96.3%, 81.94%, 1.87%, and 5.59%, respectively (Table S5)). The obtained results can be explained by the increase in the amount of the sodium alginate solution extruded from the bevel-tip capillary and are consistent with the previously reported data^23^. In addition, the wire diameter *D*_wire_ was dependent on the opening area of the bevel-tip that was proportional to the tip diameter *d* and inversely proportional to the tip angle *θ*. On the other hand, the obtained spring index *R* was within the range of approximately 1.5–5 under all studied conditions (Fig. 3e,f), indicating the formation of strong springs^24^,^25^ (the observed effects of *d, v*, and *θ* on the spring index *R* were relatively small (Table S4)). Consequently, the wire diameter *D*_wire_ can be widely controlled by varying the tip diameter *d* and, to some extent, the flow velocity *v* and tip angle *θ*; however, the spring index *R* cannot be controlled in in the same way because *R* does not depend on *d, θ*, or *v*.

Interestingly, the decrease in the flow velocity *v* causes shape transformations of the fabricated hydrogel structures from the fiber (*R* = ∞) to the spring (*R* = 1.5–5) and to the unstable structure (*R*: undefined) (Fig. 2c–e). On the other hand, the spring index *R* is not affected by the decrease in the flow velocity *v* during this process (Fig. 2e,f), suggesting that the described transformations were not continuous.

### Mechanism of formation of hydrogel microspring

**Figure 4:**
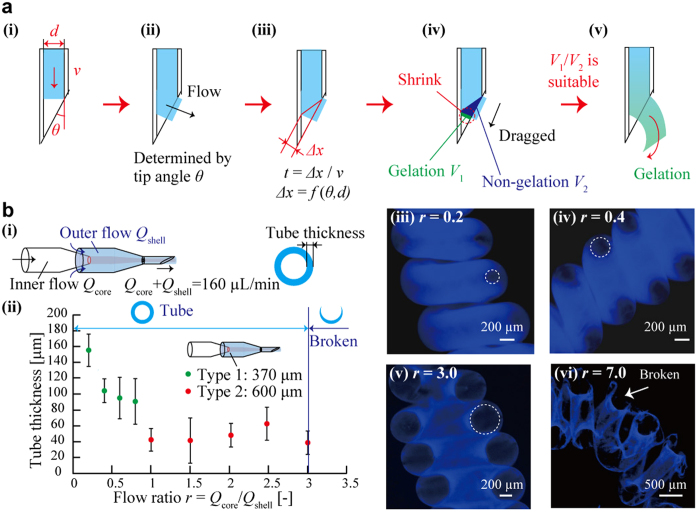
A mechanism of the microspring formation and the related applications. (**a**) A mechanism of the spring formation: (i) fabrication parameters, (ii) the flow direction is determined by the tip angle *θ*, (iii) a difference in the gelation time *t* between the two flow sides, (iv) asymmetric gelation accompanied by volumetric shrinkage (*V*_1_: gelation volume, *V*_2_: non-gelation volume), (v) a hydrogel microspring is continuously formed when the degree of asymmetric gelation *V*_1_/*V*_2_ is within a suitable range. (**b**) (i) Conditions for the tubular hydrogel microspring formation. (ii) A relationship between the tube thickness and the flow ratio *r* (the green plot: the diameter of the internal glass capillary = 370 μm, the red plot: the diameter of the internal glass capillary = 600 μm). (iii–vi) Fluorescence microscopy images of the produced tubular hydrogel microsprings. A part of the outer periphery of the tubular microsprings was broken at *r* > 3.0.

Based on the obtained results, a possible mechanism of the hydrogel microspring formation can be proposed by taking into account the gelation anisotropy around the bevel tip (Fig. 4a). First, it was assumed that the sodium alginate solution was extruded from the bevel-tip capillary with the tip diameter *d* and tip angle *θ* at the flow velocity *v* (Fig. 4a(i)). Since the tip angle *θ* determines the ejection direction of the alginate flow into the CaCl_2_ solution (Fig. 4a(ii)), the difference in the gelation starting points *Δx* = *f (d, θ*) was observed for both sides of the flow depending on the flow direction and asymmetric geometry of the bevel tip (Fig. 4a(iii)), thus producing a difference in the gelation starting time *t* = *Δx*/*v*, which was inversely proportional to the flow velocity *v* (Fig. 4a(iii)). The magnitudes of *t* and *d* determine the volumes of gelation *V*_1_ and non-gelation *V*_2_ in the cross-sectional plane of the flow around the bevel tip. As a result, the present sodium alginate in *V*_1_ shrink due to gelation, leading to an increase in the degree of the shrinkage asymmetry (*V*_1_/*V*_2_) that drags the flow to the gelation region (Fig. 4a(iv)). Finally, when the degree of the gelation asymmetry *V*_1_/*V*_2_ is within a suitable range, hydrogel microsprings are continuously formed (Fig. 4a(v)). From the described mechanism, it was concluded that the gelation at the outer periphery of the alginate flow was an important factor that affected the entire process of the hydrogel microspring formation.

### Tubular hydrogel microsprings formation

To verify the importance of gelation near the outer periphery, tubular hydrogel microsprings have been spontaneously fabricated using the bevel-tip capillary (*d* = 300 μm, *θ* = 20°) with a coaxial laminar flow. Here, the outer flow contained the sodium alginate solution, while the inner flow comprised the non-gelating propylene glycol alginate solution (Fig. 4b(i)). It was found that the inner diameter of the tubular hydrogel microspring could be controlled by changing the flow ratio *r* = *Q*_core_/*Q*_shell_ (where *Q*_core_ was the inner flow rate, *Q*_shell_ was the outer flow rate, and *Q*_core_ + *Q*_shell_ was set to a constant value of 160 μL/min; see Fig. 4b(i)). Formation of tubular microsprings was observed in the range of the flow ratio *r* between 0 and 7.0, and their thicknesses decreased with increasing flow ratio *r* from 0 to 1.0 (Fig. 4b(ii)–(iv)). At *r* > 1.0, the tube thickness reached the minimum value of approximately 40 μm (Fig. 4b(ii,v)), while at *r* > 3.0, a part of the outer periphery of the produced tubular spring was broken (Fig. 4b(ii,vi)). The obtained results indicate that hydrogel microspring formation is mainly dependent on the peripheral gelation of the outer calcium alginate shell (Fig. 4a). This results strongly support our proposal principle model. The proposed method can be used for fabricating hydrogel microsprings that are capable of encapsulating various functional materials inside their cores.

### Encapsulating functional materials

**Figure 5:**
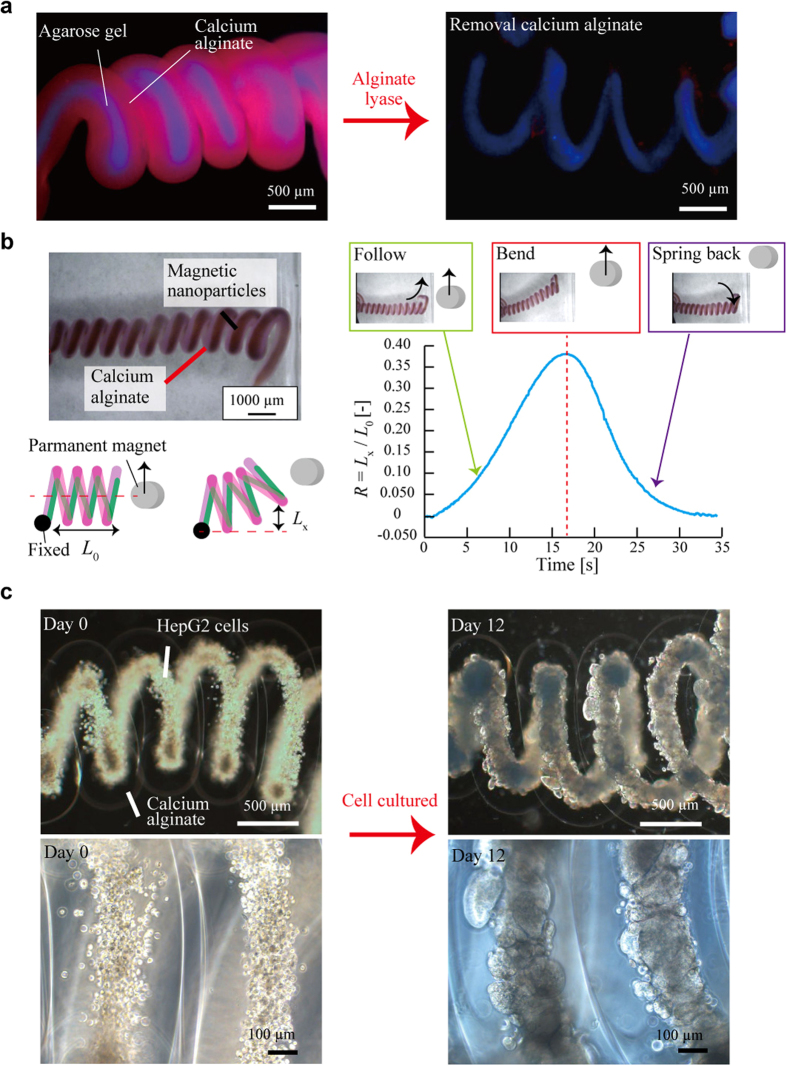
Three types of functionalized core-shell hydrogel microsprings. (**a**) An agarose-core microspring (left) and the agarose structure remained after digesting the calcium alginate shell. (**b**) A microspring encapsulating magnetic nanoparticles, which was bent by responding to the applied magnetic field and then reverted to its original form after the magnetic field was removed. (**c**) A collagen-core microspring encapsulating HepG2 cells, which were cultured for several days inside its.

Finally, in order to demonstrate the possibility of encapsulating functional materials inside the springs, the following three types of heterogeneous core-shell hydrogel microsprings were fabricated: an agarose-core microspring, a microspring encapsulating magnetic nanoparticles, and a cell-suspended collagen-core microspring. The materials encapsulated inside the microspring core were cross-linked after microspring formation by applying the appropriate gelation conditions (Table S2). First, the agarose-core microspring was produced to confirm the gelation of the inner material inside the calcium alginate shell. The agarose component was cross-linked by lowering the temperature below the gel point (around 20 °C; see Fig. 5a left). Owing to the use of alginate lyase, only the calcium alginate shell was removed via enzymatic digestion. The obtained fluorescence microscopy image showed that the agarose core maintained its spring structure due to its gelation inside the microspring (Fig. 5a right), indicating that the produced microspring could be used for manufacturing microspring-shaped functional materials by replacing agarose with other materials. Next, a magnetic nanoparticle-encapsulating microspring was formed to demonstrate its actuation properties by suspending magnetic nanoparticles in a core sodium alginate solution. As a result, the magnetic nanoparticles were trapped by the alginate hydrogel network formed after gelation (Fig. 5b left). The fabricated microspring was bent by responding to an applied magnetic field and sprang back after the field was removed (Fig. 5b right, Movie 5). The obtained results indicate that the produced spring was magnetically responsive and exhibited the properties of a mechanical spring. Finally, a collagen-core microspring encapsulating HepG2 cells was fabricated to confirm the spring capability to retain cell cultures. The cell-suspended collagen inside the microspring core was gelated after incubation at a temperature of 37 °C (Fig. 5c, left) followed by cell culturing for several days to examine possible tissue formation inside the microspring. As a result, successful tissue organization along the spring collagen core was observed (Fig. 5c, right), which was effective for mimicking tissues with similar shapes (such as Meissner’s corpuscles) and mechanically stimulable three-dimensional (3D) tissue culture platforms. We succeeded in formation of various types of functionalized core-shell hydrogel microspring by anisotropic gelation with bevel-tip capillary.

## Discussion

The mechanism of the proposed technique for producing hydrogel microsprings significantly differs from those of the previously reported microspring fabrication methods^18^,^19^,^20^,^21^. In particular, the gelation of the outer periphery of the sodium alginate flow dominates the process of microspring formation. To explain this phenomenon, three main variables (the tip diameter *d*, tip angle *θ*, and flow velocity *v*) were varied in this study. However, other parameters, such as surface wettability of a capillary, fluid viscosity and density, solution temperature, and concentration of chemicals, would also affect the properties of the produced microsprings because the entire manufacturing process involves fluid dynamics, mechanics, and chemical reactions. Hence, investigating the effects produced by these additional parameters would elucidate the related spring formation mechanism in more detail.

Using the proposed method, different types of the heterogeneous core-shell hydrogel microsprings characterized by the ability to encapsulate various functional materials were fabricated in this work, although this method relys on characteristic of sodium alginate solution. Their possible applications include micro-scale biochemical sensors and soft actuators since stimuli-responsive (to temperature^26^, light^27^, solution pH^28^ as well as to the presence of acetone^14^ and glucose^29^) hydrogels can be used as encapsulation materials for functionalized microsprings rapidly responding via large structural deformations. The complex compartmentalization of the microsprings (Figure S7) characterized by a laminar flow inside the capillary can also contribute to the optimization of their internal structure and functionality enhancement. Furthermore, microsprings composed of not only organic functional polymers^18^,^19^,^20^,^21^,^30^, but also of inorganic materials can be potentially used in new applications involving rotation motions^31^,^32^, latching^33^, THz electromagnetic metamaterials^34^,^35^ and nanoparticle composite springs^36^. We envision that our proposed method for fabricating hydrogel microsprings could open new avenues to spring-based technologies in the materials science and microengineering fields.

## Methods

### Bevel-tip capillary preparation

Three different types of ARAM perfluoroalkoxy (PFA) microtubes with inner diameters of 100 μm, 200 μm, and 300 μm were used to fabricate bevel-tip capillaries, which were subsequently cut by a tube cutter, and their tip angles were adjusted to 20–90°.

### Reagents

1.5% w/w sodium alginate solution (NaAlg; Wako, 194–13321) and 150 mM calcium chloride solution (CaCl_2_; Wako, 039–00475) were used during fabrication of hydrogel microsprings, while propylene glycol alginate (PGAlg; Wako, 165–17415) was used as an inner solution during fabrication of tubular microsprings. To produce heterogeneous core-shell hydrogel microsprings, 3% w/w agarose solution (SIGMA-ALDRICH, A2576) with a low melting point, a mixture composed of 5% v/v magnetic fluid (Ferrow Tec, EMG707) and 2.85% w/w NaAlg, and 2% w/w bovine dermal type-I collagen solution (IAC-50, KOKEN) containing HepG2 cells with a concentration of 1.0 × 10^8^ cells/mL were used as core materials. To remove calcium alginate shells, a solution containing 200 μg/mL of alginate lyase (SIGMA, A1603) in phosphate-buffered saline (×10 PBS(−), WAKO, 163–25265, was diluted by sterilized water) was utilized. To form the collagen-core microspring encapsulating HepG2 cells, a solution mixture containing 1.5% w/w NaAlg and 145 mM NaCl (Wako, 191–01665) was used as the shell flow.

### Formation of hydrogel microsprings

The bevel-tip capillary was connected to a syringe (TERUMO, 1 mL) filled with a 1.5% w/w NaAlg solution via an ethylenetetrafluoroethylene tube (VICI, 1/16″ × 0.5). Using a syringe pump (KD Scientific, LEGATO 180), the sodium alginate solution was extruded at a constant flow velocity (*v* = 0.005–0.18 m/s) into the 150 mM CaCl_2_ solution. The present NaAlg solution instantly gelates by the Ca^2+^ ions near the bevel-tip capillary; as a result, a hydrogel microspring was continuously formed. To fabricate the tubular hydrogel and heterogeneous core-shell hydrogel microsprings, a microfluidic device with parameters described in Figure S1 and Table S1 was used to create a coaxial laminar flow in the bevel-tip capillary.

## Additional Information

**How to cite this article**: Yoshida, K. and Onoe, H. Functionalized core-shell hydrogel microsprings by anisotropic gelation with bevel-tip capillary. *Sci. Rep.* **7**, 45987; doi: 10.1038/srep45987 (2017).

**Publisher's note:** Springer Nature remains neutral with regard to jurisdictional claims in published maps and institutional affiliations.

## Supplementary Material

Supplementary Information

Supplementary Movie 1

Supplementary Movie 2

Supplementary Movie 3

Supplementary Movie 4

Supplementary Movie 5
