# Supplementary material for: Functionalized core-shell hydrogel microsprings by anisotropic gelation with bevel-tip capillary
Source: Sci Rep. 2017 Apr 5;7:45987. doi: 10.1038/srep45987 (PMC5380988; doi:10.1038/srep45987)
Supplement: Supplementary Information [file srep45987-s1.pdf]

Supplementary Information

Functionalized core-shell hydrogel microsprings by anisotropic gelation with bevel-tip capillary

Koki Yoshida, Hiroaki Onoe

Center for Multidisciplinary and Design Science,
Graduate School of Integrated Design Engineering, Keio University

Table of Contents

S1. Supporting method (Figure S1, Table S1)
S2. Materials and gelation factors (Table S2)
S3. Modulus of hydrogel microspring (Figure S2, Taable S3)
S4. Scalability of produced hydrogel microsprings (Figure S3)
S5. Shape definition for hydrogel microsprings (Figure S4)
S6. Possible formation mechanisms of unstable structures and fibers (FigureS5)
S7. Success/failure of a spring formation (Figure S6)
S8. Formation conditions of hydrogel microsprings for shape analysis (Table S4)
S9. Analysis of variance on shape parameters of hydrogel microspring (Table S5)
S10 Compartmentalization of hydrogel microsprings (Figure S7)
S11. Supplementary reference
S12. Supplementary movie legends

S1. Supporting method

Reagents

A 1.5% w/w sodium alginate solution (NaAlg; Wako, 194−13321) and a 150 mM calcium chloride solution ($CaCl_2$; Wako, 039−00475) were used for the preparation of hydrogel microsprings. 5% v/v fluorescent micro beads (Life Technologies, F8810, red fluorescent, 0.2 μm; F 8805, blue fluorescent, 0.2 μm; or F8811, yellow-green fluorescent, 0.2 μm) were encapsulated in the sodium alginate solution for the hydrogel and agarose gel visualization. Propylene glycol alginate (PGAlg; Wako, 165−17415) was used as an inner solution for manufacturing tubular microsprings.

To fabricate heterogeneous core-shell hydrogel microsprings, we used 3% w/w agarose with a low melting point (SIGMA-ALDRICH, A2576), 5% v/v magnetic fluid (Ferrow Tec, EMG707) containing 2.85% w/w NaAlg, and 2% w/w bovine dermal type-I collagen (IAC-50, KOKEN) containing HepG2 cells with a concentration of around 1.0×10^8 cells/mL as core materials.

To remove the calcium alginate shell, it was digested by a solution containing 200 μg/mL of alginate lyase (SIGMA, A1603) in phosphate buffered saline (×10 PBS(−), WAKO, 163−25265, was diluted by sterilized water).

To fabricate a collagen-core microspring encapsulating HepG2 cells, a solution mixture containing 1.5% w/w NaAlg and 145 mM NaCl (Wako, 191−01665) was used as the shell flow. NaAlg was sterilized with a 0.22-μm sterilizing filter (Millex, SLGV033RS). The $CaCl_2$ solution and the NaCl solution were autoclaved. In addition, Dulbeco’s modified eagle medium (DMEM, SHIGUMA, D5796), fetal bovine serum (FBS, SELBORNE, FB−1365), and penicillin-streptomycin solution (antibiotics, AB, SGUMA−ALDRICH, P4458) were obtained. HepG2 cells (RIKEN BRC CELL BANK, RCB No. 1886) were maintained in the culture medium composed of the DMEM + 10% FBS + 1% AB aqueous mixture saturated with 5% of CO_2 gas at a temperature of 37 °C.

Microfluidic devices with a coaxial laminar flow

Three different types of the microfluidic devices with a coaxial laminar flow were utilized for adjusting the velocities of the outer flow and shell flow (Table S1). In particular, two microfluidic devices were fabricated by assembling a glass capillary with an outer diameter of 1.0 mm and inner diameter of 0.6 mm (NARISHIGE, G−1), which was adjusted to 370 μm (type 1) by a puller (NARISHIGE, PC−10) or 600 μm (type 2), a rectangular glass capillary with an outer diameter of 1.4 mm and inner diameter of 1.0 mm (Vitrocom, 8100−100), and a connector created using a 3D printer (Keyence, AGILISTA; see Figure S1a). The remaining device (type 3) was fabricated by assembling a glass capillary with an outer diameter of 1.5 mm and inner diameter of 0.9 mm, and a connector with an inner diameter of 2.5 mm (Figure S1b).

Fabrication setup for tubular hydrogel microsprings: Two types of the microfluidic devices with a coaxial laminar flow (types 1 and 2) were used. The type 1 connector was utilized at $Q_{core}/Q_{shell} < 1.0$, and the type 2 device was used at $1.0 < Q_{core}/Q_{shel}$. To produce tubular hydrogel microsprings, the following two solutions were prepared: a non-gelation solution of 2% w/w PGAlg for the core stream, and a pre-gel

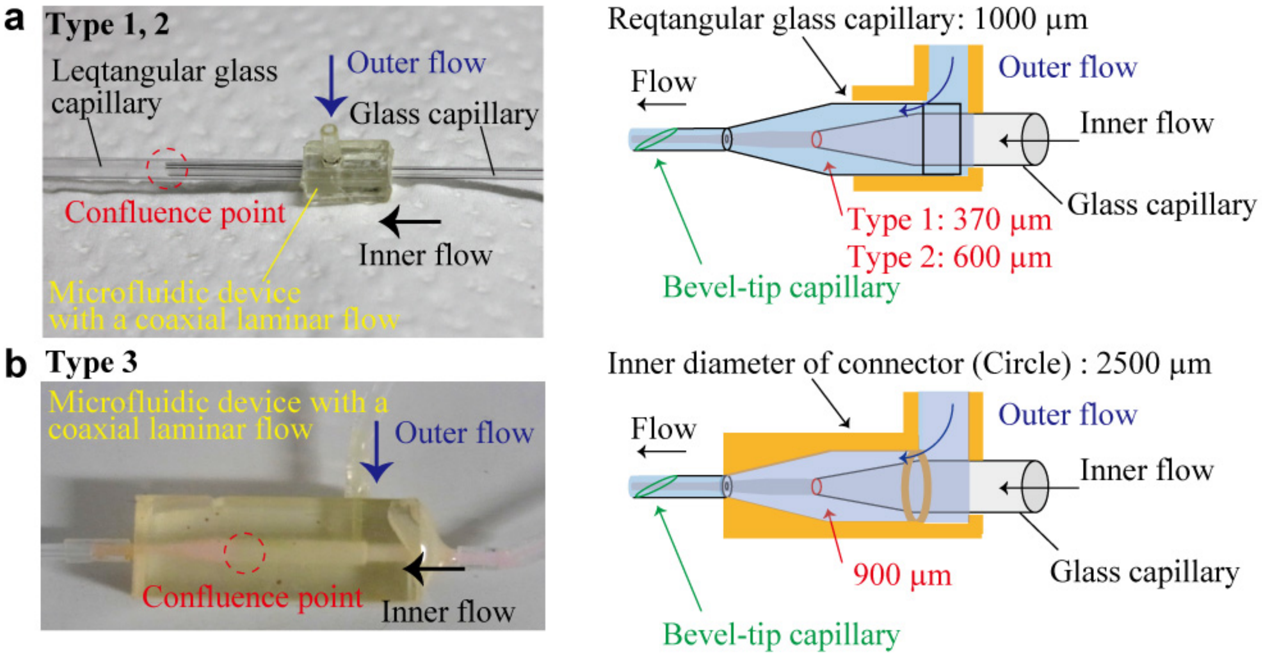

Figure S1 Photographs of the studied microfluidic devices with a coaxial laminar flow. (**a**) types 1 and 2, and (**b**) type 3.

Table S1 Parameters of the utilized connectors and core materials (all dimensions are in mm).

Connector type	Outer capillary	Inner capillary	Core material
1	□ square 1×1	○ outer: 1, inner: 0.37	PGAlg, Agarose, Collagen
2	□ square 1×1	○ outer: 1, inner: 0.60	PGAlg
3	○ inner: 2.5	○ outer: 1.5, inner: 0.90	NaAlg with magnetic nanoparticles

solution of 1.5% w/w NaAlg for the shell stream. The diameter of the bevel-tip capillary was 300 μm, and the tip angle was 20°.

Fabrication setup for heterogeneous core-shell hydrogel microsprings

To fabricate an agarose-core microspring, we used the type 1 connector and the following two solutions: a 3% w/w agarose pre-gel solution for the core stream and a pre-gel solution of 1.5% w/w NaAlg for the shell stream. The outer flow rate was 185 μL/min, and the inner flow rate was 15 μL/min. The tip diameter was 300 μm, and the tip angle was 20°. The agarose component was cross-linked by lowering the temperature below the gelling point (around 20 °C). After the microspring formation, it was transferred to an ice-cooled 150 mM $CaCl_2$ solution with a temperature of approximately 4 °C. The pre-gel agarose solution was warmed by hot water to a temperature of 100 °C before use, while the entire experimental setup was warmed using an infrared lamp (110 V, 250 W, IWASAKI ELECTRIC, IR100–110V250WRH).

To produce a microspring encapsulating magnetic nanoparticles, the type 3 connector was used. A pre-gel solution mixture containing 5% v/v magnetic fluid and 2.85% w/w NaAlg was used for the core stream, and a pre-gel solution of 1.5% w/w NaAlg was utilized for the shell stream. The outer flow rate was 240 μL/min, and the inner flow rate was 40 μL/min. The tip diameter was 300 μm, and the tip angle was 20°. During magnetic actuation, one microspring end was immobilized by a tweezer. A permanent magnet (Magfine, Neodymium magnet, circular, φ20×6) was moved away from the other microspring end at a constant speed of 1 mm/s. The magnitude of the initial magnetic field applied to the microspring was approximately 0.3 T. The microspring bending motion was analyzed using a special video analysis software (Keyence, VW−H2MA).

To fabricate a collagen-core microspring encapsulating HepG2 cells, we used the type 1 connector and the following two solutions: a type-I collagen solution containing HepG2 cells with a concentration of around 1.0×10^{8} cells/mL for the core stream and a pre-gel solution mixture containing 1.5% w/w of NaAlg and 145 mM of NaCl for the shell stream. Before loading solutions into the microfluidic device, both the device and bevel-tip were filled with ethanol for sterilization. During loading, the outer flow rate was 85 μL/min, and the inner flow rate was 10 μL/min. The tip diameter was 200 μm, and the tip angle was 20°. After the spring was formed, it was immediately transferred to a dish filled with the culture medium and then cultured at a temperature of 37 °C in water saturated with 5% of CO_2 gas.

Observation of hydrogel structures

The obtained hydrogel structures were characterized by an inverted fluorescence microscope (OLYMPUS, IX73P1−22FL/PH). Microspring sizes were measured using special image-processing software (OLYMPUS, cellSens).

Shape characterization of hydrogel microsprings

The diameters of the first three turns of six randomly chosen microsprings were measured, and their corresponding error bars were computed as standard deviations.

S2. Materials and gelation factors

The utilized materials and gelation factors are summarized in Table S2. After the formation of heterogeneous core-shell hydrogel microsprings, the inner materials immediately gelled by applying the corresponding gelation factors.

Table S2 Materials and gelation factors.

Materials	Gelation factors
Sodium alginate	Ca^{2+}
Propylene glycol alginate	None
Agarose	Gel point ≤ 20 °C
Collagen	Gel point ≥ 37 °C

S3. Modulus of hydrogel microspring

To measure the spring constant of our hydrogel microspring (D_{wire} = 300 μm). We built a force measurement setup using two glass capillaries (a glass capillary (NARISHIGE, G-1) and a quarts capillary (WJM-Glass, outside diameter of capillary: 0.1 mm, length: 80 mm, thickness of the capillary wall: 0.01 mm)). These capillaries were fixed by micromanipulators (NARISHIGE, M-152) respectively. Each end of the hydrogel microspring was bonded to the glass capillary and the quarts capillary respectively (Figure S7a). By pulling the microspring with the glass capillary, a displacement of the quarts capillary Δx was measured by a digital microscope (Keyence, VH-5500). The movement of glass capillary was controlled by a micromotion stage (SHIGUMA-KOKI, HPS80-50X-M5). A tensile load of spring p was calculated by $p = (3E_q I_q \Delta x) / x_q^3$ (Parameters were showed in Table S5, Figure S7b), where E_q is young's modulus of the quarts capillary, I_q is secondary moment area and x_q is load point. An extension of hydrogel microspring, x, was calculated by $x = X - \Delta x$ (X is a moving distance of the glass capillary). A modulus of hydrogel microspring k was determined $k = 3\times10^{-3}$ N/m by a graph of p vs. X (Figure S7c).

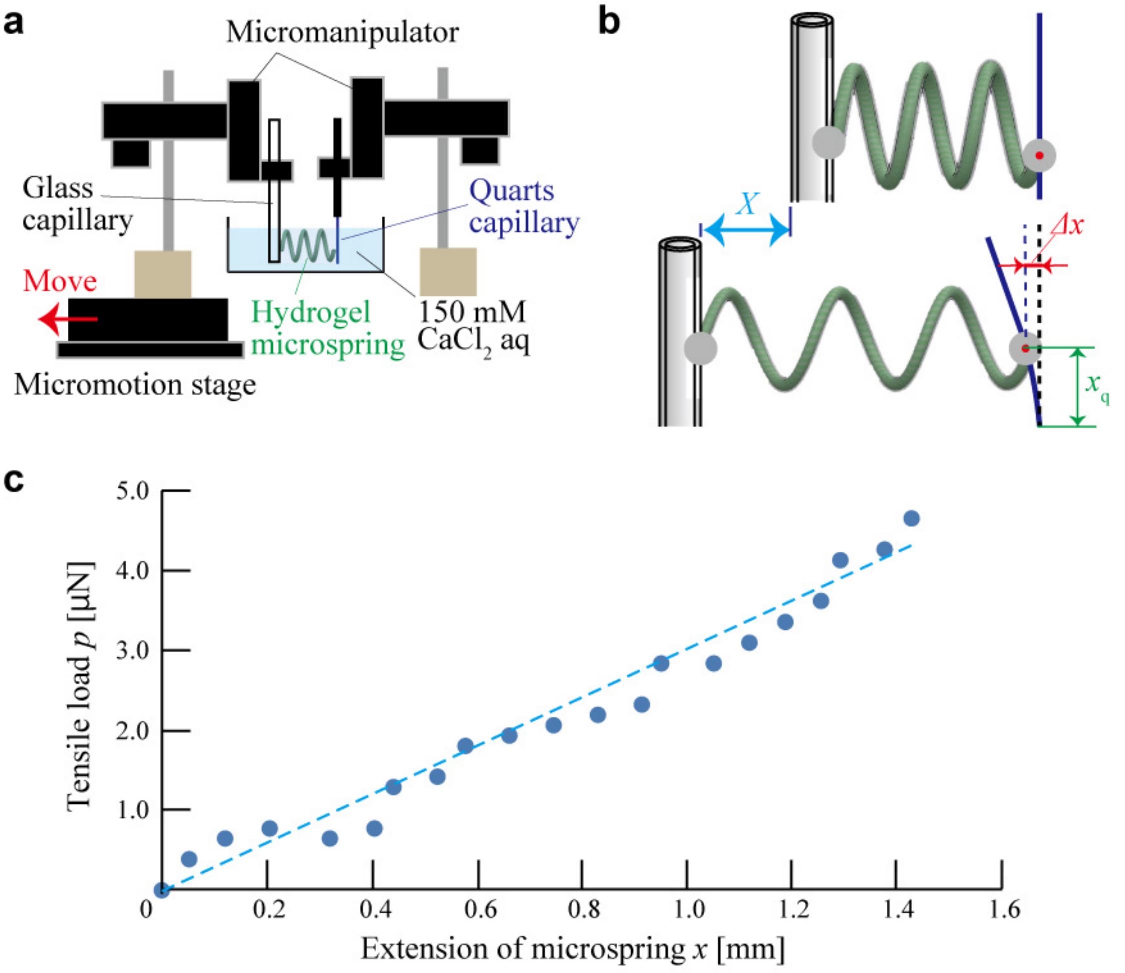

Figure S2 Modulus of hydrogel microsprings. (**a**) measurement set up, (**b**) measured parameters, (**c**) tensile load p vs. extension of microspring x.

Table S3 Parameters for calculation of modulus.

Young's modulus E_q	74 GPa
Load point x_q	57 mm
Secondary moment area I_q	6.8×10^{-18} m^4

S4. Scalability of produced hydrogel microsprings

We fabricated hydrogel microsprings of various sizes by using bevel-tip capillaries with different diameters (d = 70–900 μm; see Figure S5a). The smallest hydrogel microspring was characterized by D_{wire} = 62 μm and D_{spring} = 188 μm (Figure S5b), while the largest hydrogel microspring exhibited D_{wire} = 584 μm and D_{spring} = 2790 μm (Figure S5c).

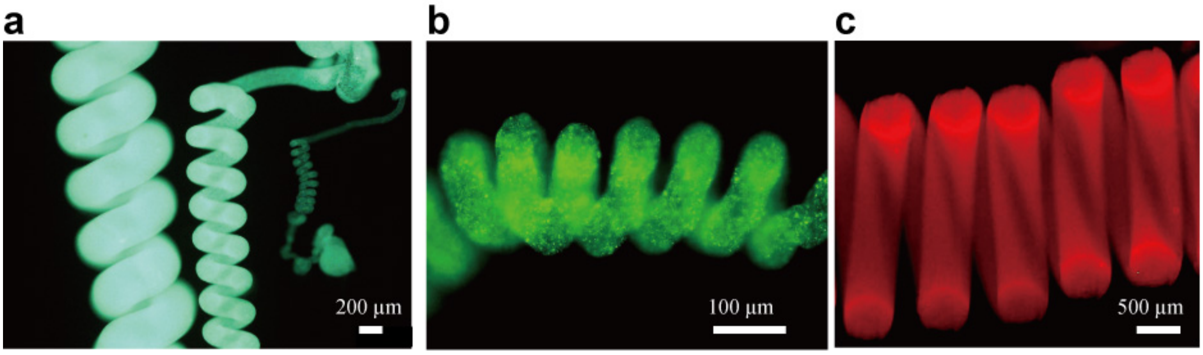

Figure S3 Scalability of produced hydrogel microsprings. (**a**) Size variations of the produced hydrogel microsprings. (**b**) The smallest hydrogel microspring: D_{wire} = 62 μm and D_{spring} = 188 μm. (**c**) The largest hydrogel microspring: D_{wire} = 584 μm and D_{spring} = 2790 μm.

S5. Shape definition for hydrogel microsprings

We defined “spring” as an object that had more than three turns and was characterized by the variations of the radius r and pitch p of each turn that differed from those of the previous turns by less than a factor of 2. The fiber was straight line, while the unstable structure was defined as the shape that was neither a spring nor a fiber.

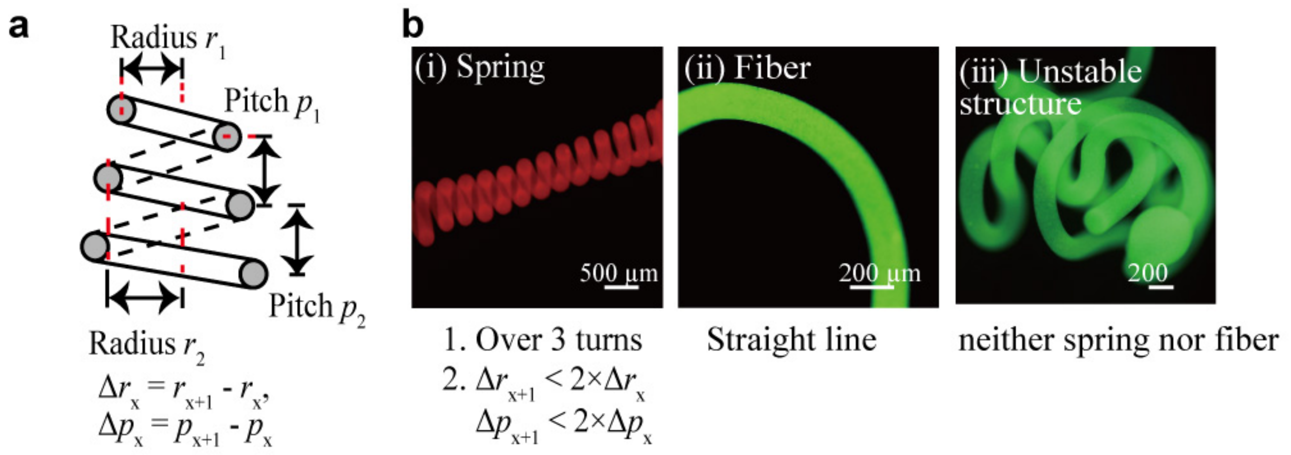

Figure S4 Definitions of the produced hydrogel structures. (**a**) Parameters, which were used to determine whether the fabricated structure was a spring. (**b**) The obtained hydrogel structures were divided into the following three types: (i) springs, (ii) fibers, and (iii) unstable structures.

S6. Possible formation mechanisms of unstable structures and fibers

When the flow velocity v is too low, unstable structures are formed (Figure S4a). It is assumed that the gelation of sodium alginate occurs inside the bevel-tip capillary rather than near the tip. Thus, other complex factors (such as the friction between the gelated alginate and the inner capillary wall) may affect the microspring formation mechanism, which results in the creation of unstable structures.

On the other hand, the main reason for the fiber formation at high flow velocities is the fact that the flow velocity v is greater than the diffusion speed of Ca^{2+} ions in the calcium chloride solution (Figure S4b). As a result, the volume of gelated calcium alginate structure near the bevel-tip is not large enough for anisotropic gelation, and a fiber is formed instead of a spring.

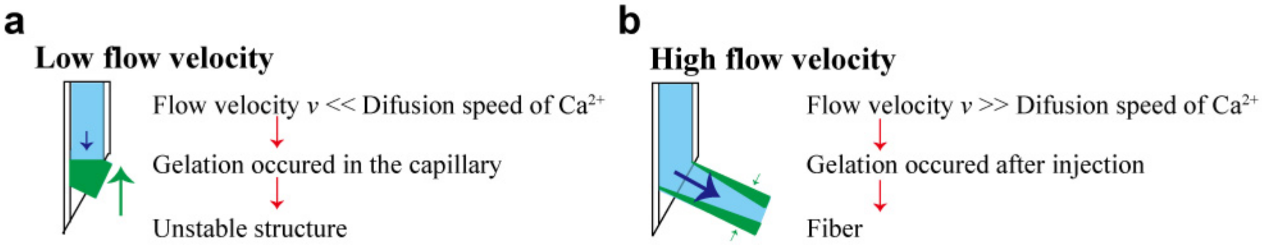

Figure S5 A relationship between the produced hydrogel structure and the flow velocity v. (**a**) When the flow velocity is low, an unstable structure is formed. (**b**) When the flow velocity is high, a fiber is produced. The ratio between the flow velocity v and the diffusion speed of Ca^{2+} ions determines the shape the ultimate structure.

S7. Success/failure of a spring formation

The detailed experimental results obtained in this study are provided in Figures 2c–e. Five hydrogel structures were obtained at each condition, and different frequencies of the successful spring formation were denoted in Figures S3a–c by the quarter-filled circles (1 spring after 5 trials), half-hilled circles (2 springs after 5 trials), three-quarter filled circles (3 springs after 5 trials), and fully filled circles (4 or 5 springs after 5 trials). When spring formation was not observed at a given condition, the resulting hydrogel structure was determined to be a fiber or an unstable structure. After comparing the numbers obtained for each structure, the corresponding plots were divided into the following four regions: an "only fibers" region, a "springs or fibers" region, a "springs or unstable structures" region, and an "only unstable structures" region (Figure S3d).

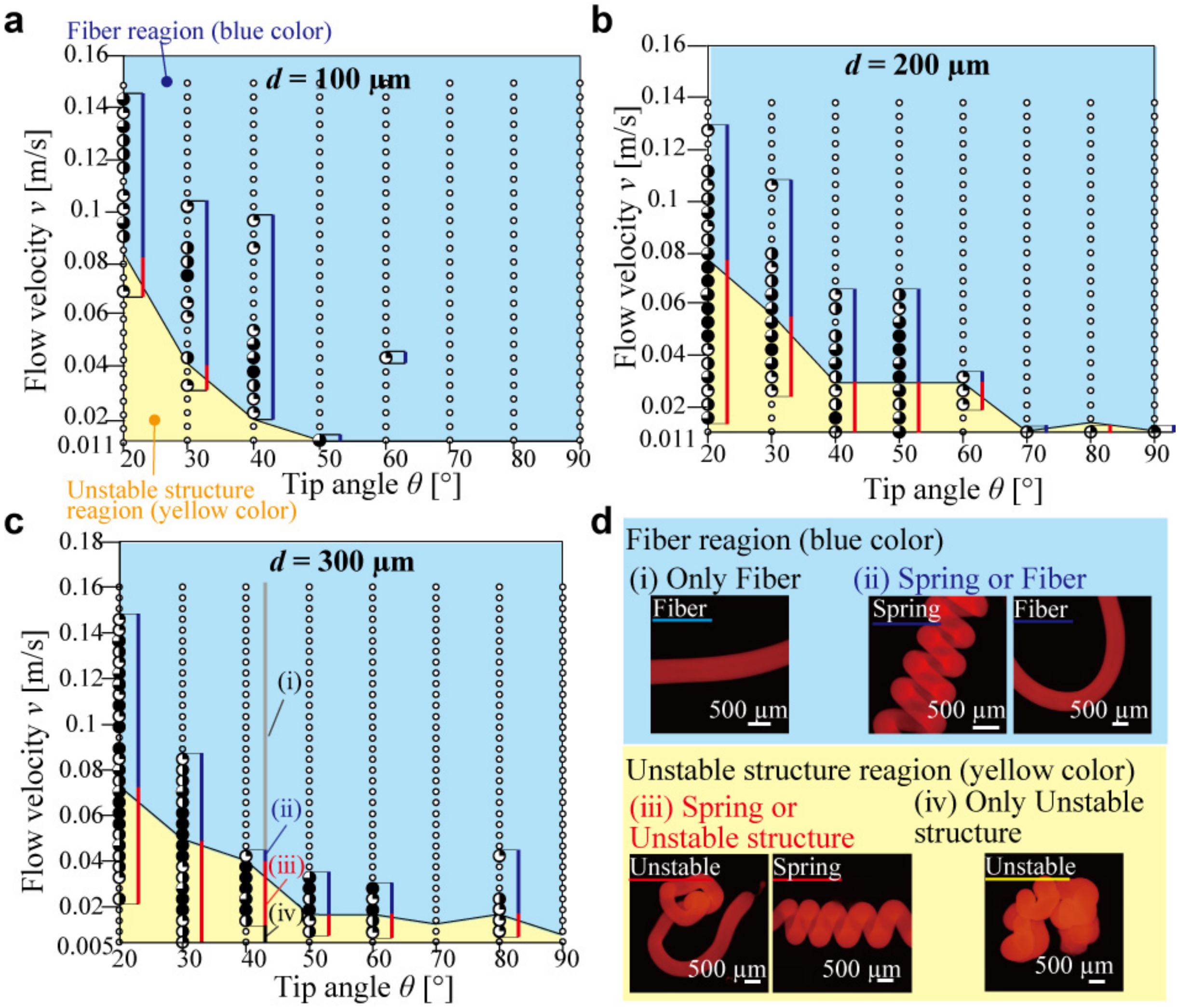

Figure S6 Success/failure of spring formation. (**a**)–(**c**) Detailed parameters obtained for the produced hydrogel structures by varying the flow velocity *v* (a: *d* = 100 μm, b: *d* = 100 μm, and c: *d* = 300 μm). (**d**) Descriptions of the following four regions on the obtained plots: (i) only fibers, (ii) springs or fibers, (iii) springs or unstable structures, (iv) only unstable structures.

S8. Formation conditions of hydrogel microsprings for shape analysis

The formation conditions of the produced microsprings for shape analysis (Figires 2h-k) are summarized in Figures 2h–k. Table S3 lists the values of the flow velocity v obtained at different tip angles and tip diameters: $v = 90.19\times10^{-3}$ m/s (d = 100 μm, θ = 20°), $v = 84.88\times10^{-3}$ m/s (d = 100 μm, θ = 30°, 40°), $v = 58.36\times10^{-3}$ m/s (d = 200 μm, θ = 20–40°), and $v = 42.44\times10^{-3}$ m/s (d = 300 μm, θ =20–40°).

Table S4 Parameters of the microspring shape analysis.

Tip diameter d [μm]	Tip angle θ [°]	Flow velocity v [m/s]
100	20	0.090
	30,40	0.085
200	20–40	0.058
300	20–40	0.042

S9. Analysis of variance on shape parameters of hydrogel microspring

We performed the analysis of variance for investigating the influences of the tip diameter *d*, the tip angel θ and the flow velocity *v* on the shape of hydrogel microsprings (the wire diameter D_{wire} and the spring index *R*)[1]. Table S4 shows a contribution ratio of each factor, and * was marked when a risk was less than 1% (*f*: degree of freedom; *S*: type III sum of squares; *V*: standard devision; F_0: F ratio; *S'*: net sum of squares; ρ value: significance probability; ρ: contribution ratio; *e*: error; *T*: corrected total). Since the influence of the flow velocity *v* on the wire diameter D_{wire} is much smaller than the influence of the tip diameter *d*, the variance analysis of tip angle θ and the wire diameter D_{wire} was performed ignoring the influence of the flow velocity *v*.

Table S5 Results of the variance analysis conducted for the main microspring shape parameters.

D_{wire} (*v*, *d*)

Source	*f*	*S*	*V*	F_0	*S'*	ρ value	ρ (%)
v	2	9340	4670	30.1	9030	0.000	1.87*
d	2	465000	233000	1500	465000	0.000	96.3*
v × *d*	4	1240	309	2.00	618	0.111	0.13
e	45	6990	155				1.71
T	53	483000					

D_{wire} (θ, *d*)

Source	*f*	*S*	*V*	F_0	*S'*	ρ value	ρ (%)
θ	2	39200	19600	23.2	37500	0.000	5.59*
d	2	552000	276000	326	551000	0.000	81.94*
θ × *d*	4	42300	10600	12.5	38900	0.000	5.79*
e	45	38100	846				6.68
T	53	672000					

R (*v*, *d*)

Source	*f*	*S*	*V*	F_0	*S'*	ρ value	ρ (%)
v	2	8.07	4.04	11.9	7.39	0.000	24.99*
d	2	2.03	1.01	3.00	1.35	0.060	4.57
v × *d*	4	4.28	1.07	3.17	2.93	0.022	9.90
e	45	15.2	0.338				60.54
T	53	29.6					

R (*θ*, *d*)

Source	f	S	V	F_0	S'	ρ value	ρ (%)
θ	2	3.03	1.51	23.2	2.65	0.001	18.25*
d	2	0.883	0.442	326	0.509	0.105	3.50
$\theta \times d$	4	2.22	0.556	12.5	1.48	0.029	10.15
e	45	8.40	0.187				68.10
T	53	14.5					

S10. Compartmentalization of hydrogel microsprings

To investigate various patterns formed inside the produced hydrogel microsprings, the following compartmentalized hydrogel microsprings were obtained by creating patterned laminar flows: a core-shell hydrogel microspring, two different types of double-layered hydrogel microsprings, and two different types of dual-core hydrogel microsprings (Figure S6). The corresponding laminar streams were injected at a constant flow rate into the calcium chloride solution.

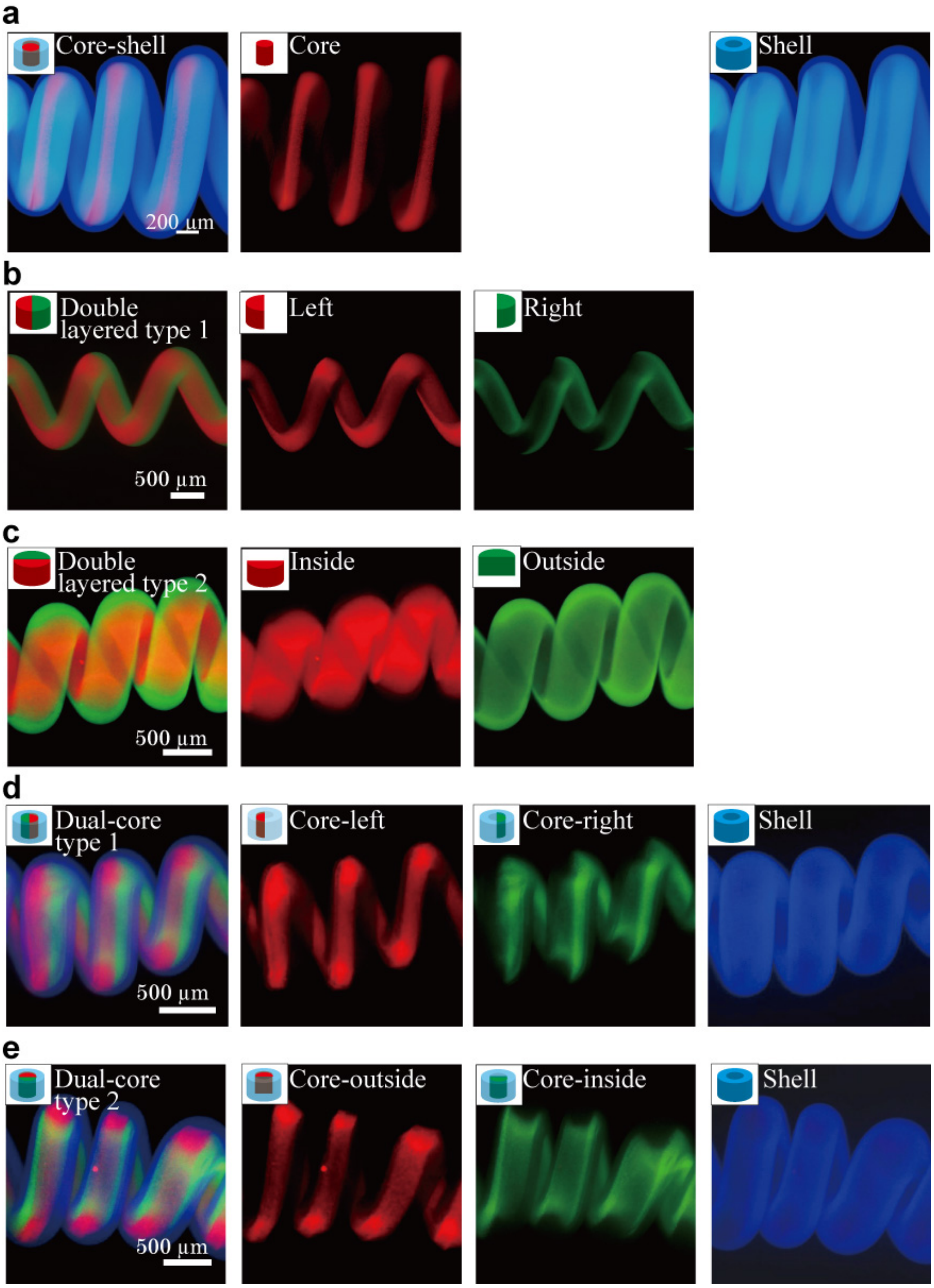

Figure S7 Compartmentalized hydrogel microsprings. (**a**) a core-shell hydrogel microspring, (**b**) a double-layered hydrogel microspring (type 1), (**c**) a double-layered hydrogel microspring (type 2), (**d**) a dual-core hydrogel microspring (type 1) and (**e**) a dual-core hydrogel microspring (type 2).

S11. Supplementary reference

[1] P. R. Krishnaiah, *Handbook of Statistics 1 Analysis of Variance*, 1980

S12. Supplementary movie legends

Supplementary movie 1: Forming hydrogel microspring

Supplementary movie 2: Formation of fiber

Supplementary movie 3: Formation of spring

Supplementary movie 4: Formation of unstable structure

Supplementary movie 5: Magnetic actuation
